# Perspectives on inadequate preparation and training priorities for physicians working with sexual minority youth

**DOI:** 10.5116/ijme.615c.25d3

**Published:** 2021-10-27

**Authors:** Lindsay A. Taliaferro, Joanna Mishtal, Veenod L. Chulani, Tiernan C. Middleton, Meagan Acevedo, Marla E. Eisenberg

**Affiliations:** 1 Department of Population Health Sciences, University of Central Florida, Orlando, Florida, USA; 2 Department of Anthropology, University of Central Florida, Orlando, Florida, USA; 3 Section of Adolescent Medicine, Phoenix Children's Hospital, Phoenix, Arizona, USA; 4 Department of Pediatrics, University of Maryland Medical Center, Baltimore, Maryland, USA; 5 rnold Palmer Hospital for Children, Orlando Health Regional Medical Center, Orlando, Florida, USA; 6 Division of General Pediatrics and Adolescent Health, University of Minnesota, Minneapolis, Minnesota, USA

**Keywords:** Adolescent, sexual minority, pediatric, family medicine, qualitative

## Abstract

**Objectives:**

To understand pediatric and family medicine residents' and practitioners' perceived ability to work with lesbian, gay, bisexual, and queer (LGBQ) youth, assessment of their prior educational experiences, and recommendations for medical training to better prepare physicians to provide quality care to this population.

**Methods:**

We conducted semi-structured individual interviews with 24 pediatric/family medicine residents (n=20) and practicing physicians (n=4) in the U.S. Recorded interviews were professionally transcribed. Data were analyzed using Grounded Theory and qualitative content analysis approaches.

**Results:**

Most physicians did not feel adequately prepared to provide quality care to LGBQ youth, and many who felt knowledgeable obtained their knowledge from on-the-job experiences of caring for LGBQ patients. Findings regarding physicians' recommendations for implementing a formal training program revealed three themes: (I) medical school training (implemented earlier in medical school within a structured program as part of the normal curriculum), (II) training content (LGBQ-specific health needs, self-awareness of implicit biases, interviewing techniques, and resources), and (III) training strategies (panels of LGBQ patients, role-playing/standardized patients, and online modules).

**Conclusions:**

Understanding physicians' assessment of abilities and recommendations for training improvements based on their experiences is important for advancing the quality of healthcare for LGBQ youth. Guidance came mostly from residents who recently completed medical school. Thus, their perspectives are especially useful to improve medical education and, ultimately, the care provided to LGBQ youth. Findings suggest a multi-pronged approach that offers several training modalities encompassing individual, intrapersonal, and institutional/systemic/community levels can improve medical school curricula on caring for LGBQ youth.

## Introduction

Young people who identify as a sexual minority (LGBQ; lesbian, gay, bisexual, or queer) experience significant health disparities, compared to heterosexual youth.^1^^,^^2^ However, medical school and residency programs spend minimal time providing clinical training and education to trainees regarding delivering affirmative, inclusive, culturally competent, and high-quality care to sexual minority youth. Many physicians feel less comfortable and poorly prepared to counsel and care for sexual minority youth and report gaps in training and education.^3^^-^^7^ For example, in one study, approximately 40% of physicians received no formal training on working with sexual minorities.^8^ A review of LGBQ-related content in curricula of 176 medical schools showed that the content varies and often remains fair to poor quality.^9^ In another study, 75% of physicians in a medical school providing training on caring for LGBQ patients found this education inadequate.^4^ Investigators also found that almost half of first-year medical students in a large U.S. sample expressed some explicit bias, and most expressed some implicit bias against LGBQ individuals.^10^ Researchers attribute such negative attitudes, in part, to medical education curricula and culture,^8^ which leaves clinicians feeling unprepared to provide culturally competent care to LGBQ youth.^11^

Inadequate and/or poor-quality training for health care providers is linked to delayed care for LGBQ patients. In addition, adolescence represents a unique time in the life-course marked by sexual identity development^12^^,^^13^ and increased involvement in risk behaviors.^14^^,^^15^ LGBQ youth, in particular, demonstrate an increased risk of involvement in certain health-risk behaviors, mental health problems (e.g., substance misuse, suicidality), and homelessness due to experiences of stigma, prejudice, and discrimination that create hostile and stressful social environments.^1^^,^^16^^-^^18^ Physicians who care for adolescents and young adults (AYAs; aged 10-24) need additional training that specifically addresses the needs of sexual minority youth,^3^^-^^5^ beyond simply risk for HIV and other sexually transmitted infections (STIs), and prepares them to meet their unique healthcare needs.

Professional associations have proposed clinical guidelines, recommendations, and best practices for providing care to LGBQ patients.^19^^,^^20^ In addition, curricula and training resources for clinicians have been developed.^21^^-^^24^ However, no mandate exists requiring programs to incorporate training within undergraduate, graduate, or continuing medical education on working with sexual minority youth.^25^ Research shows that clinicians caring for AYAs do not feel fully prepared or comfortable providing care to sexual minorities.^3^^-^^7^ While studies show that weaknesses in perceived preparation and abilities result from inadequate training,^4^^,^^8^^,^^9^ data are lacking regarding what pediatric and family medicine physicians believe they need to feel more competent to provide quality care to LGBQ AYAs and how they would prefer medical training on this topic be structured. Curriculum development appears to be often driven by what a select few individuals believe is necessary, rather than by what pediatric and family medicine physicians-in-training and in-practice say they need to feel more comfortable and competent.^24^ Qualitative interviews represent an optimal method of eliciting new ideas from physicians, including recommendations for training development and implementation, because participants have the opportunity to explain nuanced perceptions and experiences. Further, individual interviews produce in-depth data, in physicians' own words, that can facilitate understanding of training needs directly from the target population. To address gaps in the literature, we sought to understand pediatric and family medicine residents' and practitioners' perceived ability to work with LGBQ AYA patients, assessment of their prior educational experiences, and recommendations for medical training to better prepare physicians to provide quality care to LGBQ youth using individual interviews. Findings move the field forward by describing concrete ideas to improve physician training.

## Methods

### Study design, participants, and setting

This research used a qualitative design to glean in-depth insights from healthcare providers regarding their experiences and training needs working with LGBQ AYAs. We conducted semi-structured individual interviews between July 2017 and March 2018 with 24 pediatric and family medicine residents and practicing pediatricians and family medicine physicians in the U.S. whose patient populations include AYAs (see Table 1 and Table 2 for participant characteristics). We excluded physicians who were not in pediatrics or family medicine because providers in pediatric and family medicine provide primary care for the vast majority of youth (aged 10 to 24).^26^ Participants were recruited using purposeful and snowball sampling.^27^ We recruited physicians through the national Academic Pediatric Association's Continuity Research Network (CORNET), referrals from residency directors around the U.S. known to the study team, and snowball sampling with other physician participants. Participants were emailed the informed consent form, had an opportunity to ask the interviewer any questions, and provided verbal consent prior to commencing the interview. The University of Central Florida Institutional Review Board approved the study.

### Data collection

Our data collection followed a systematic approach through the development and use of a 24-item interview guide, including probing questions. We developed the interview instrument through a review of the research literature and consultation with members of the study team with expertise in the health of sexual minority youth. We pre-tested the questions with eight physicians (2 paediatricians, one family medicine physician, two pediatric residents, and three family medicine residents) to ensure phrasing was appropriate and well-worded,^27^ and revised questions based on pre-test feedback. The interview guide topics included questions regarding physician perceptions of their knowledge and ability to work with LGBQ AYA patients; anti-LGBQ biases; awareness of LGBQ healthcare issues; and training needs related to providing quality care to LGBQ AYAs. In this paper, we focus on data related to the following questions: (1) How knowledgeable and prepared do you feel about providing quality healthcare to LGBQ youth?; (2) What training, if any, have you had that has helped you feel prepared and comfortable providing quality care to LGBQ youth?; (3) What aspects of the training you had were most helpful and why?; and (4) What training interventions could have better prepared you to provide quality care to this population?

Interviews were conducted via telephone by a medical student experienced and trained in qualitative research methods. All participants provided verbal consent and agreed to be audio-recorded. Interviews lasted 20-40 minutes. Participants received a $35 electronic gift card at the conclusion of the interview for their time.

**Table 1:** Resident and practicing physician sample demographic characteristics (N = 24)

Demographic characteristics		Frequency (%)
Assigned sex at birth		
	Female	15 (62)
	Male	9 (38)
Race/ethnicity		
	White	18 (75)
	Asian	4 (17)
	Black/African American	2 (8)
Sexual orientation		
	Heterosexual	21 (88)
	Gay	1 (4)
	Lesbian	1 (4)
	Bisexual	1 (4)
Physician type		
	Resident	20 (83)
	In-Practice	4 (17)
Specialty		
	Pediatrics	17 (71)
	Family Medicine	7 (29)

### Data analysis

Recorded interviews were professionally transcribed verbatim. Team coder training was employed to minimize the threat of bias in the analysis. Each transcript was coded in its entirety, and coders identified themes irrespective of where the data appeared in the transcript. We generated and refined a codebook of themes through an iterative process, wherein themes were identified as common explanations expressed by multiple participants. The coding process was inductive and followed the "dynamic and fluid process" as informed by the Grounded Theory (we did not follow Grounded Theory as a method to generate a theory,^28^ but rather as a way to conduct a careful thematic analysis of participants' standpoints) as well as qualitative content analysis approaches.^29^^,^^30^ These approaches allow for both predetermined, a priori codes to be explored, as well as the emergence of inductive, not previously considered factors or explanations,^31^^,^^32^ while the topic of "training" in medicine was a predetermined topic of inquiry. We also identified explanations that were emergent, for example, panels of LGBQ patients, online modules. We proceeded from open to axial coding. To minimize bias and ensure inter-coder consistency in data analysis, two members of the study team individually coded a transcript (authors TM and JM) to generate themes, seek consensus on codes and interpretations, and resolve minor thematic differences to generate a preliminary codebook.^31^ The codebook was further refined during the coding process in team meetings. Completion of the coding process generated a detailed thematic dataset. All transcripts were coded by two members of the study team (TM and JM). We used NVivo 11 software to perform a data-driven thematic analysis. The sample size permitted thematic saturation, which means explanations became repetitive (no further new themes emerged) for a given interview question or topic category.^29^^,^^33^^,^^34^

## Results

Most physicians in our sample did not feel adequately prepared to provide quality care to LGBQ youth. Among those who felt knowledgeable, many indicated this knowledge came from on-the-job experiences of learning to care for LGBQ patients, specifically watching their attending physician work with LGBQ patients.

"My attending was the one who actually told me about how to ask these questions, and also how to be more sensitive and compassionate, and be a patient listener. Make sure you don't have like a weird expression on your face. I mean, all subtle things like that, that I think were really useful, which had come to her by experience. And I think looking at her, observing her doing it, was really helpful to me." (Participant 13)

Or, physicians acquired knowledge through their direct clinical interactions with sexual minority youth.

"It has really just been based on experience, and unfortunately, learning through trial-and-error. [I] think a lot of it has just been based on experiences with individual patients. But I'm sure there's [laugh] a better way to do it besides trial-and-error with every patient you meet." (Participant 9)

As noted by Participant 20, "more formal training" would enhance knowledge and perceived competence, as opposed to learning "in bits and pieces." Findings regarding physicians' recommendations for implementing a formal training program revealed three themes: (I) medical school training, (II) training content, and (III) training strategies. We chose representative quotes throughout the results to illustrate these themes.

### Theme I. Medical school training: earlier and integrated approach needed

Participants indicated they learned more about providing care to LGBQ youth during residency than medical school, and they thought formal training should be implemented earlier in medical school.

"It would have given me more time to practice because I was on different rotations with different patient populations. [W]hen you're a medical student, you're given a little more leeway to make mistakes, to have to correct yourself. And I might have felt more comfortable doing that [laugh] rather than when I'm supposed to be your provider." (Participant 19)

"[W]e should start training about this right from medical school, because once you come to residency and all of a sudden start hearing about new things, it just becomes a little awkward." (Participant 13)

When implementing training in medical school, participants suggested delivering a structured program, particularly during education on adolescent medicine.

"I feel like a good moment to introduce the topic would probably be in medical school. I think introducing that training as part of the introduction to adolescent medicine on a pediatric clerkship would be really beneficial because it's early on and kind of starts the thought process of how this population has unique needs, and they may have different healthcare needs as well." (Participant 9)

"I think we should have learned more about it, like with didactics, in the first or second year, just so everyone could be on the same page with terminology and that sort of thing, and what the AAP recommends, and what their stance is on counseling and talking to parents and that sort of thing." (Participant 5)

"I kind of feel like this ought to be (…) part of educating people in medical school about adolescent medicine. And I think not only exposure to it, but having some curriculum behind it to teach the types of things that not only adolescents but everyone in the LGBT community faces that can lead to bad health outcomes if you're not open to it or not attuned to it." (Participant 8)

In addition, some participants indicated they did not think education about caring for LGBQ youth should be a stand-alone module. Instead, this education should occur as a normal part of the curriculum.

"[I] t's so difficult because you say, 'Well, it would be nice to have more detailed training about how best to care for this population,' but then you don't want to make it so much that they're some kind of other group that it becomes almost like stereotyping or profiling in a way." (Participant 9)

"I think it can be added (…) as 'This is the norm. You will meet people who are this, like this, and this is what they need,' just like we would do for any other minority or like socioeconomic status. Just as a normal part of the curriculum versus a pull-out specialized section." (Participant 10)

### Theme II. Training content: specific and comparative

Physicians described needing training that addressed four areas. To understand: (1) LGBQ-specific health needs/issues they should address ("specific needs"), (2) implicit biases they may have regarding LGBQ youth ("inherent biases"), (3) interviewing techniques ("a little script"), and (4) "resources" available to share with sexual minority youth patients.

(1) "Specific needs". Generally, physicians expressed a lack of training regarding sexual health and other areas specific to this population, and they thought a training should address LGBQ-specific healthcare issues.

"Really the different health issues and stuff that we should make sure we're addressing [with LGBQ youth]. So we're not missing anything." (Participant 12)

"[I] would definitely be interested in learning more about their specific needs and what other things they might be looking for when they come to see the doctor, besides what their presenting medical issue might be." (Participant 9)

Providers expressed the need for LGBQ-specific health training in three areas: risk factors, psychosocial issues, and screenings.

"I want to know the statistics about basically any differences in mental healthcare, ways in which to help these patients approach friends and family who might be disapproving of their lifestyle decisions, and then anything I need to know regarding sexual health practices and how to best approach that." (Participant 14)

"In my own personal experience, the medical side of it is easy. It's the psychosocial support side that's critical and huge, and this massive gap right now. [W]hen something like this is put together in terms of actually educating us to meet the medical and psychosocial needs of our patients, it would be really (…) important to build that curriculum taking into consideration what a large number of patients have experienced and have indicated they would have wanted or should have received." (Participant 22)

"[A]lso just the kind of things about, like mental health concerns, social issues, home environment issues, bullying and things like that." (Participant 11)

"We didn't get a lot of training on screening. Like health screenings, or guideline-based evidence for this population. Like what to treat, what to screen, what vaccines – things like that." (Participant 4)

(2) "Inherent biases". Some participants demonstrated self-awareness by communicating the possibility that they possess implicit biases against sexual minorities.

"I still feel like I have a long way to go in terms of my inbred assumptions and working past those." (Participant 22)

"I think no matter how hard I try to be accepting and open-minded and caring, I think there's always going to be, just because of my experience and where I'm coming from, I'm always going to have some biases that I need to be able to – I wouldn't say look past, but just kind of be aware of, when I'm trying to care for these individuals." (Participant 9)

Based on this awareness, a learning objective recommendation involved helping physicians recognize and address personal biases.

"If there's any kind of inherent biases that we may have as practitioners that we could try to avoid, that could be helpful." (Participant 14)

(3) "A little script". Communicating effectively with LGBQ patients represented an important training area desired by participants. To ensure they provide quality care, physicians requested training on interviewing techniques and questions.

"[H]ow to ask the questions. Or how to just open the conversation. I feel like that's an area where I struggled in the beginning as an attending. How to just bring up the subject. Just more interviewing techniques more than anything. I think almost providing (…) a little script on how to open up these conversations. How to make it easy to talk to them about it, especially when they notice that they are resistant to disclose it." (Participant 21)

"[T]echniques for talking to teenagers and how to frame certain questions. I kind of learned from experience to always ask, 'Are you interested in men, women, or both?' Because I've had answers that surprised me, so I've just kind of learned that on my own. But maybe different ways of framing these kinds of questions and making teens feel comfortable during an interview." (Participant 11)

"We're not really trained with (…) how to approach a patient after we get that information that they're lesbian, gay, whatever, bisexual. I think we're trained to get that information, but then after that, I think we're not really trained like what to do with it." (Participant 1)

(4) "Resources". A significant gap in physicians' training involved knowing resources to provide LGBQ patients who might need information and/or support. Thus, participants suggested training programs include information on specific resources they can provide this patient population.

"[S]he asked for resources from me, and I was stunned and I had no clue what to do." (Participant 13)

"I’m very comfortable as far as having discussions with them about being affirmative and positive and trying to support them in that way. But I think that in order to have a little bit more meat behind those words, I need to have some better knowledge of follow-through and about options they have in the community, or people in the community they can talk to, or things like that. Where it’s not just about giving them words of encouragement and positive affirmation, but also following through with something tangible that they can then look into in their own time and feel supported in that way as well.” (Participant 9)

### Theme III. Training strategies: face-to-face, simulated, and virtual

Physicians consistently suggested that useful training should include: (1) panels of LGBQ patients, (2) role playing/standardized patients, and (3) online modules as methods for learning about providing quality care to LGBQ youth.

(1) Panels of LGBQ patients. Given the unique patient population addressed in this study, providers indicated wanting to learn from patients themselves.

“[I]t would be great if I can hear from a panel of individuals who are in the LGBTQ community. I want to hear from them regarding what their experiences were when they came to the physician’s office, and how they think we could change, and I think could become more sensitive. And that’s when I think I’ll be most comfortable, instead of just imagining and cooking in my head regarding what they would need.” (Participant 13)

“And there’s really not a lot of you know just sort of training. A local conference that says, ‘Here’s what’s under your nose every day. And here’s what they want you to be.’ Not what we think we should be, but what the kids want us to be. Right? What the kids need us to be. We don’t get that enough.” (Participant 22)

“And then also maybe just tips and getting the perspective from somebody who is homosexual, and things that made them feel more comfortable. Like tools we could use to make them feel more comfortable.” (Participant 12)

(2) Role-playing/standardized patient scenarios. To ensure concepts learned through didactic presentations are well-understood, clinicians suggested including role-playing exercises and standardized patient experiences within a training program.

“I think the biggest thing for training-wise would be walking through – so actually taking time to develop different visits and walking through that visit in different scenarios. (…) to actually practice the wording of that, or provide a framework of this is a suggested way to approach that, and how to counsel different patients in those scenarios. I think what would be the most helpful is kind of the mechanics of doing that through a visit.” (Participant 17)

“Role-play. (…) making them as realistic but awkward as possible. Because I think having a set way to get yourself into or out of a situation or a conversation – if there’s like a good way to use verbiage to transition into a more [laugh] suitable conversation, I think I would like to have that in my toolkit.” (Participant 18)

“(…) modeling the conversation. I got to have a conversation with a 12-year-old boy this year who asked me about anal sex. Well, ain’t nobody ever talked to me about that. Boy, it would be really nice for us to learn, so that we can respond to children in a knowledgeable fashion, instead of having no clue.” (Participant 22)

 (3) Online modules. Providers suggested training on providing care to LGBQ youth include an online component.

“I feel online modules as well as live training (…). We have similar trainings for CPR training and things like that. Why can we not have trainings for things like this?” (Participant 13)

“I think an online module or an online forum board that helps you walk through some of those concepts.” (Participant 17)

**Table 2:** Characteristics of participants

Participant number	Specialty	Resident/ Practitioner	Sexual Orientation	Gender Identity	Race
1	Family Medicine	1^st^ Year Resident	Lesbian	Female	White
2	Family Medicine	3^rd^ Year Resident	Heterosexual	Female	Black
3	Family Medicine	Resident, year unknown	Heterosexual	Male	White
4	Family Medicine	3^rd^ Year Resident	Heterosexual	Female	White
5	Pediatrics	1^st^ Year Resident	Heterosexual	Female	Asian
6	Internal Medicine/ Pediatrics	2^nd^ Year Resident	Heterosexual	Female	White
7	Internal Medicine/ Pediatrics	2^nd^ Year Resident	Heterosexual	Male	White
8	Family Medicine	1^st^ Year Resident	Heterosexual	Male	White
9	Pediatrics	2^nd^ Year Resident	Heterosexual	Female	Asian
10	Pediatrics	2^nd^ Year Resident	Bisexual	Male	White
11	Pediatrics	4^th^ Year Resident	Heterosexual	Female	White
12	Pediatrics	4^th^ Year Resident	Heterosexual	Female	White
13	Pediatrics/Medical Genetics	4^th^ Year Resident	Heterosexual	Female	Asian
14	Pediatrics	2^nd^ Year Resident	Heterosexual	Male	White
15	Pediatrics	1^st^ Year Resident	Gay	Male	White
16	Pediatrics	1^st^ Year Resident	Heterosexual	Female	Black
17	Pediatrics	3^rd^ Year Resident	Heterosexual	Male	White
18	Internal Medicine/ Pediatrics	3^rd^ Year Resident	Heterosexual	Female	White
19	Pediatrics	4^th^ Year Resident	Heterosexual	Female	White
20	Family Medicine	2^nd^ Year Resident	Heterosexual	Male	Asian
21	Pediatrics	Practicing Physician	Heterosexual	Female	White
22	Pediatrics	Practicing Physician	Heterosexual	Female	White
23	Pediatrics	Practicing Physician	Heterosexual	Female	White
24	Family Medicine/ Sports Medicine	Practicing Physician	Heterosexual	Male	White

## Discussion

Reviews of medical education curricula related to caring for sexual minorities indicate that contents are variable, patchy, and generally inadequate. Therefore, understanding physicians’ personal assessment of abilities and recommendations for medical training improvements is important to advance the quality of healthcare for this population. We sought to address gaps in the literature regarding training structure, content, and strategies desired among pediatric and family medicine residents and practicing physicians on providing quality care to LGBQ AYAs. Consistent with previous research, our findings show that physicians’ education in the area of LGBQ youth care remains inadequate.^4^^,^^35^^,^^36^ Furthermore, our findings add to the extant literature by showing that physicians obtained most of their knowledge at a later point in their education through graduate medical education and clinical practice, including learning from their attending physicians and personally providing care to LGBQ patients. This finding highlights the need to implement training improvements at an earlier stage of medical education. Participants acknowledged the value of clinical interactions with LGBQ patients, and previous research found that medical students with increased clinical exposure to LGBQ patients demonstrated more positive attitudes toward and greater knowledge of LGBQ health concerns than those with little or no clinical exposure.^6^ Still, participants also expressed concern about not feeling fully prepared to provide high-quality care to this population and “learning through trial-and-error” and in “bits-and-pieces”.

Taken together, our findings suggest that medical school curricula on caring for LGBQ youth would benefit from a multi-pronged approach that offers several training modalities. Overall, physicians suggested implementing more formal training during medical school, in which instructors address LGBQ-specific health needs, self-awareness of implicit biases toward LGBQ youth, interviewing techniques with this population, and information on resources for LGBQ patients. Suggested teaching modalities include panels of LGBQ patients, role-playing/standardized patients, and online modules.

Physicians recommended opportunities to learn directly from patients such as panel discussions and presentations that include LGBQ patients. These represent patient-centered methods of learning and provide clinicians the opportunity to learn directly from the patient population for whom they will care, which improves learning more than solely relying on opportunistic patient contacts in clinical settings.^37^ Patients are experts in their experiences and may enhance learning of real-world medicine by sharing their perspectives with trainees.^38^^-^^40^ Involving patients in planning medical curriculum could further improve patient care, satisfaction, and health outcomes.^41^ Community engagement of marginalized populations, such as sexual and gender minorities, is especially important and helpful in cultivating improbable discussions, yielding innovative insights, and building relations for future collaborations and interventions to improve healthcare for these populations.^40^

In addition, physicians in our sample recognized the value of utilizing other forms of exposure to LGBQ AYAs, including role-playing simulations and virtual online modules. Role-playing with feedback allows clinicians to practice skills and enhances their capacity to address diverse adolescent health issues.^42^^,^^43^ This teaching method might be especially helpful in educating physicians to discuss topics they find uncomfortable or challenging,^44^^,^^45^ including LGBQ healthcare issues.^46^ Online modules also represent a teaching method that could be effectively implemented within medical school curricula on LGBQ health.^22^ Interactive online medical education modules have shown similar effects for learning and may better facilitate the application of knowledge than traditional lectures.^47^

### Implications for medical education and future research

We believe the results that emerged from this study present a useful starting point from which to consider concrete improvements to medical education curricula. We propose that medical schools consider a multi-pronged approach by developing, implementing, and evaluating required training programs in LGBQ healthcare that encompass three levels:

1. Individual: From pre-clinical to clinical years, this component would include individual reflection exercises on implicit biases, progressing to peer discussions and sharing sessions with providers experienced in caring for LGBQ adolescent patients to stimulate discussion, exchange knowledge, challenge biases, and ultimately empower patient-centered care of LGBQ youth. (Attitudes/Affective Component)

2. Interpersonal: To develop a range of communication skills for clinical interactions from pre-clinical to clinical years, this element would start with online training modules using virtual patients, panel presentations with LGBQ patients, role-playing simulations, and exposure to LGBQ patients in encounters in the clinical years. (Behavioral/Skills Component)

3. Institutional/Systemic/Community: This component would progressively include content on minority stress, health disparities and LGBQ health issues, and building knowledge and exposure to local and online resources available to support LGBQ patients and address LGBQ issues. (Lends a socio-ecological and strengths-based lens; Knowledge/Cognitive Component)

We propose that incorporating LGBQ-related health themes longitudinally through multiple years of education is particularly important,^24^ given students may need time to recognize and overcome implicit biases and develop requisite communication skills prior to their translation into effective patient-centered care delivered in everyday clinical settings. In addition, while it is necessary to base LGBQ-related healthcare education on research data that capture the specific needs of this population, integrating this topic into formal training within a range of standard curricula and rotations, including pediatrics, adolescent medicine, sexual and reproductive healthcare, obstetrics and gynecology, and general practice, rather than isolating LGBQ care into a siloed stand-alone area of learning, will be important. Testing scenarios also should go beyond mentioning a patient’s sexuality only when this aspect of a person’s identity is tied with his/her/their sexual activity or risk for HIV and other STIs.

These improvements may serve as important future research foci to conduct continuous quality improvement evaluation. In addition, future research should conduct efficacy and effectiveness studies using experimental and longitudinal research designs to determine which recommended strategies are most impactful in improving medical students’ knowledge, skills, attitudes, and behavior related to engaging in best clinical practices when interacting with LGBQ AYAs. Further, investigators should consider conducting research to determine the combination of strategies and the amount of exposure to curricula content that prove most effective in changing behavior and practices. Ultimately, future research should examine effects of improved training in LGBQ AYA healthcare on LGBQ patients’ perceptions of clinical interactions with trained clinicians, engagement in healthcare, including needed follow-up or specialty care, and physical and psychosocial health outcomes.

### Limitations

Findings should be interpreted in the context of some sample limitations. Our sample was predominantly female, white, and heterosexual. Their perspectives may differ from those of other physicians. Also, the sample included a greater proportion of residents than physicians in-practice. Nevertheless, our data showed consistency in responses between residents and practicing physicians, regardless of year in residency or years in practice, and perspectives of residents may be particularly useful for considering medical education improvements because of their more recent experience with medical education curricula. Findings from this qualitative interview-based study were not meant to be generalizable to the general population.

## Conclusions

Providing patient-centered care requires training physicians to remain mindful, informative, and empathetic in their goals of partnership, solidarity, and collaboration with patients.^48^ This approach is essential in the care of LGBQ youth.^49^ Cultural competence related to providing quality care to LGBQ youth would allow physicians to provide compassionate and understanding care, while also demonstrating competence regarding the role of sexual identity in patients’ overall health and well-being. Resources exist to help clinical educators incorporate curricula on caring for LGBQ youth into their training programs.^21^^,^^24^^,^^50^ Our findings represent the real-world voices and experiences of frontline physicians who care for LGBQ youth, and what these physicians need in their own words. In particular, guidance on curriculum content and strategies that would help physicians feel better prepared to provide care to LGBQ youth came mostly from residents who recently completed medical school and found their training on this topic inadequate. Thus, their perspectives are especially useful to improve medical education and, ultimately, the care provided to LGBQ youth.

### Conflict of Interest

The authors declare that they have no conflict of interest.
